# Haemolytic uremic syndrome: diagnosis and management

**DOI:** 10.12688/f1000research.19957.1

**Published:** 2019-09-25

**Authors:** Neil S. Sheerin, Emily Glover

**Affiliations:** 1 National Renal Complement Therapeutics Centre, Institute of Cellular Medicine, Newcastle University and Biomedical Research Centre, Newcastle-upon-Tyne NHS Foundation Trust, Newcastle-upon-Tyne, UK

**Keywords:** Haemolytic Uraemic Syndrome, Thrombotic microangiopathy, Complement

## Abstract

The thrombotic microangiopathies (TMAs) are a group of diseases characterised by microangiopathic haemolysis, thrombocytopenia, and thrombus formation leading to tissue injury. Traditionally, TMAs have been classified as either thrombotic thrombocytopenic purpura (TTP) or haemolytic uremic syndrome (HUS) based on the clinical presentation, with neurological involvement predominating in the former and acute kidney injury in the latter. However, as our understanding of the pathogenesis of these conditions has increased, it has become clear that this is an over-simplification; there is significant overlap in the clinical presentation of TTP and HUS, there are different forms of HUS, and TMAs can occur in other, diverse clinical scenarios. This review will discuss recent developments in the diagnosis of HUS, focusing on the different forms of HUS and how to diagnose and manage these potentially life-threatening diseases.

## Introduction

The term thrombotic microangiopathy (TMA) refers to a group of diseases characterised by endothelial dysfunction and the formation of platelet- and fibrin-rich thrombi in small blood vessels. As the thrombus forms, there is consumption of platelets and mechanical disruption of erythrocytes, which leads to the typical haematological manifestations of TMA, thrombocytopenia and microangiopathic haemolytic anaemia (schistocyte [fragment] formation, raised lactate dehydrogenase, and reduced haptoglobin). Previously, the TMAs were classified according to their clinical presentation with neurological symptoms predominating in thrombotic thrombocytopenic purpura (TTP) and acute kidney injury (AKI) in haemolytic uremic syndrome (HUS). However, there is significant overlap in the clinical features of TTP and HUS and the diseases are now classified based on aetiology. TTP is caused by an inherited or acquired (inhibitory autoantibody) defect in ADAMTS13 activity, a circulating protease responsible for the degradation of ultra-large von Willebrand factor multimers. In the absence of ADAMTS13, these multimers accumulate on the surface of endothelial cells, leading to the formation of platelet-rich thrombi.

The classification of HUS is more complex, as a pattern of clinical disease consistent with HUS can occur in a wide range of clinical scenarios. Most cases (90%) occur following infection with a Shiga toxin-producing bacteria, either enterohaemorrhagic *Escherichia coli* (STEC) or *Shigella dysenteriae*, and typically affect children. The remaining 10% of cases have traditionally been grouped together as atypical HUS. However, as our understanding of the pathogenesis of HUS has increased over the last 20 years, it has become clear that atypical HUS is not one but many different diseases with similar clinical features. A TMA with a pattern of disease consistent with HUS can occur because of inherited disorders, acquired disease states (including malignancy and autoimmune disease), in pregnancy, in severe hypertension, and in response to drugs. The situation is further complicated by the interaction between inherited predisposition to disease and environmental triggers. As a consequence, there is still no universally accepted classification of HUS. In this review, we will, as far as possible, classify HUS according to its aetiology but accept that this may not capture the complexity of the disease pathogenesis.

## HUS associated with infection

Infections are the most common cause of HUS. In addition, infection, for example influenza ^1^, may act indirectly as a trigger for the development of a TMA in patients with a genetic predisposition to disease.

### Shiga toxin-induced HUS

HUS associated with infection with a Shiga toxin-producing bacteria (usually STEC HUS) is one of the commonest causes of AKI in children but can occur at any age ^2^. Although adaptive immune responses can be identified after exposure to enteropathogenic *E. coli*, it is unclear whether these are protective ^3^, and the higher rate of infection in children may be due to more frequent exposure. The annual incidence of STEC HUS is approximately 0.7 cases per 100,000 population, with the highest incidence in the summer months. In Europe and North America, *E. coli* O157 is the serotype most commonly associated with HUS; however, this appears to be changing, with fewer cases of O157-associated HUS and increasing numbers of cases due to other serotypes reported in some countries ^4^. In parts of Asia, *S. dysenteriae* type 1 infection is the most common precipitant.

***Clinical features***. Abdominal pain, diarrhoea (bloody in 60%), and vomiting typically occur 3 days after ingestion of bacteria. Approximately 10% of patients exposed to bacteria will then develop HUS. The development of HUS is influenced by both pathogen- (inoculum size, pathogen strain, and type of toxin produced) and host-related factors (microbiome ^5^, antimotility and antibiotic drug use, inflammatory response, and possible genetic factors ^6^). In 5–10% of patients with STEC HUS, there will be no preceding history of diarrhoea, highlighting the importance of microbiological investigation of all patients with a TMA irrespective of history ^7^.

Renal involvement is present in most cases and dialysis is required in 50%. Renal recovery after 1–2 weeks is usual, and end-stage renal disease (ESRD) after the initial presentation is uncommon. Neurological involvement is the most frequently reported extra-renal manifestation with seizures and reduced consciousness levels reported in up to one-third of cases ^8, 9^. Other sites that can be affected include the intestinal tract and pancreas, eyes, and heart. The mortality rate in the acute phase of the disease is approximately 2–5% ^10^ in STEC HUS but is higher when HUS follows *Shigella* infection ^11^. Long-term renal sequelae are common, with hypertension and chronic kidney disease (CKD) occurring in 25–40% of patients ^12, 13^.

***Pathogenesis***. Shiga toxin-producing *E. coli* can colonise the intestine of healthy animals and can enter the food chain if infected meat or other food products are consumed. Pathogenic *E. coli* closely associate with the gastrointestinal mucosa and toxin translocates through the epithelium into the circulation where it binds to circulating erythrocytes and leukocytes. The toxin, which comprises one A and five B subunits, binds to globotriaosylceramide (Gb3) on the surface of target cells. It is taken up by endocytosis and is then retrogradely transported to the endoplasmic reticulum ^14^. The A subunit inhibits protein synthesis, disrupting cell function and ultimately leading to cell death. The toxin also incites an inflammatory response, the net effect being to establish a pro-thrombotic state within the microvasculature ^15^. The concentration of cell surface Gb3 is particularly high in the kidney, not only on the endothelium but also on podocytes and the tubular epithelium. This may explain the susceptibility of the kidney to injury, but other factors, such as high renal blood flow, may also contribute. Gb3 is also found in other tissues, including the central nervous system, that are affected by STEC HUS.

***Diagnosis and management***. Faecal culture should be performed in all patients with a TMA irrespective of whether there is a history of colitic illness. Diarrhoea may have settled by the time of presentation, but bacteria can still be isolated; therefore, stool or rectal swab should still be sent. PCR can be performed on these samples to detect the presence of Shiga toxin genes. Positive serology for antibodies against toxin-producing *E. coli* serotypes is supportive of a diagnosis of STEC HUS, although it is not routinely used.

Currently, there is no evidence that treatments other than supportive care, including dialysis and ventilator support as required, improve the outcome of patients with STEC HUS. Plasma-based therapy is no longer recommended for the treatment of STEC HUS and may in fact be harmful ^16^.

The role of antibiotics is controversial and their use has traditionally been avoided because of concerns that antibiotic treatment leads to more Shiga toxin release, increasing the likelihood and severity of STEC HUS ^17^. The effect of antibiotics appears to be both antibiotic and *E. coli* strain dependent. The β lactams and trimethoprim/sulfamethoxazole have been reported to increase the risk of developing HUS ^17– 19^. Although fluoroquinolones increase toxin production *in vitro*, they do not appear to worsen disease. Some antibiotics, such as macrolides and fosfomycin, reduce toxin synthesis and may reduce the risk of HUS ^20, 21^. When antibiotics are required, they can be used, but the choice of antibiotic should be guided by their potential effect on toxin release. In contrast, antibiotic use in patients with HUS due to *Shigella* appears to be safe. Trials of toxin-binding resins have failed to show a benefit.

A role for complement in the pathogenesis, and therefore a rationale for anti-complement therapies, has been proposed. Preclinical studies in rodent models of STEC HUS have suggested that complement activation is involved in disease development ^22– 24^; however, animal models of STEC HUS have limitations, and an effect of complement inhibition was not seen in a primate model. In patients with STEC HUS, there is evidence of complement activation ^25^ and C3 levels can be low. In addition, there may be some patients with STEC HUS who also have a defect in complement regulation, and this combination is associated with a poor prognosis ^26– 28^. These observations have led to the use of eculizumab, a humanised monoclonal antibody raised against complement protein C5, in patients with STEC HUS. There have been reports of patients with STEC HUS, particularly in the presence of severe disease, responding to eculizumab ^29, 30^. However, STEC HUS is a self-limiting disease and therefore these reports have to be interpreted with caution. The use of eculizumab following the outbreak of STEC HUS in central Europe due to *E. coli* O104 yielded conflicting reports, with some suggesting benefit ^31^ and others none ^16^. At present, there is insufficient evidence to support the use of eculizumab in patients with STEC HUS outside of a clinical trial ^32^. Randomised trials in France (ECULISHU, NCT02205541) and the UK (ECUSTEC, ISRCTN 89553116) are investigating the potential benefit of eculizumab in STEC HUS and will provide much-needed evidence in this area.

### Pneumococcal HUS

This normally affects children with severe pneumococcal infection, for example pneumonia or empyema, when it complicates approximately 0.5% of cases ^33^. The exact mechanism of TMA is unknown, but the production of neuraminidase by *Pneumococci* appears to be important, although other mechanisms have been proposed ^34^. Neuraminidase strips sialic acid from cell surface glycoproteins, exposing the cryptic Thomsen-Friedenreich (T) antigen, which is recognised by naturally occurring IgM antibodies ^35^. This leads to the activation of platelets and endothelium, which may be responsible for the TMA and also explains why these patients have a positive Coombs (DAT) test.

Pneumococcal pneumonia is the most common preceding infection, but TMA can develop in association with meningitis, empyema, sinusitis, and otitis media. Because of the underlying disease, children are often unwell, with 75% requiring dialysis, and extra-renal manifestations occur frequently ^36^. The mortality rate is higher than with STEC HUS. Treatment is supportive. The role of plasma exchange is uncertain ^37^, and, although a response to eculizumab has been reported ^38, 39^, evidence for its use is limited.

### HUS occurring in association with other infections

A TMA was a relatively common complication of HIV infection (incidence 2–7%) ^40^ but is seen less frequently following the introduction of highly active anti-retroviral therapy (HAART) (<1%) ^41^. TMA was associated with high viral load, low CD4 count, and opportunistic infection ^41^ and may be due to a direct effect of the virus on the renal endothelium. Although there have been reports of a response to eculizumab, this is started in combination with HAART and it is therefore difficult to determine which intervention is having the major effect ^42^. TMA has been associated with a range of other infections ( Table 1), although for most whether they are a direct cause or a trigger for the TMA remains uncertain.

**Table 1.** Infections associated with the development of a thrombotic microangiopathy.

Shiga toxin-associated diarrhoeal illnesses
***Escherichia coli O157:H7, O26, O80, O91, O103, O104, O111, O121,*** ***O145*** ***Shigella dysenteriae type 1***
Non-Shiga toxin-associated diarrhoeal illnesses
**Norovirus** ***Clostridium difficile*** ***Campylobacter upsaliensis***
Respiratory tract infections
***Streptococcus pneumoniae*** ***Haemophilus influenzae*** ***Bordetella pertussis***
Other bacterial infections
***Fusobacterium necrophorum***
Viral infections
**HIV** **Cytomegalovirus** **Epstein Barr virus** **Varicella zoster** **Influenza** **Hepatitis A** **Hepatitis C** **Coxsackie B** **Dengue virus** **Human herpes virus 6** **Parvovirus B19**
Parasitic infections
***Plasmodium falciparum***

## Atypical HUS

### Complement-mediated atypical HUS

In the 1990s, excessive activation of complement was identified as a cause of atypical HUS ^43^. An inherited or acquired defect in the control of complement activation can now be found in approximately 60% of cases. In addition, a role for complement activation has been suggested when a TMA occurs in specific clinical scenarios, although the contribution of complement activation in these situations is less clear. In this review, we will consider atypical HUS occurring in the presence of a defined defect in complement regulation as a distinct entity and consider “secondary” TMAs separately.

***Clinical features***. Complement-mediated atypical HUS predominantly affects the kidney, but, as with other forms of HUS, extra-renal manifestations are also seen in approximately 20% of cases, neurological disease being the most common. Older reports suggest a poor prognosis, with up to 50–60% of patients dying or progressing to ESRD within 1 year of presentation. This is influenced by underlying genetic factors, as patients carrying mutations in complement Factor H have a worse prognosis and those with mutations in CD46 have a milder disease.

**Figure 1.**
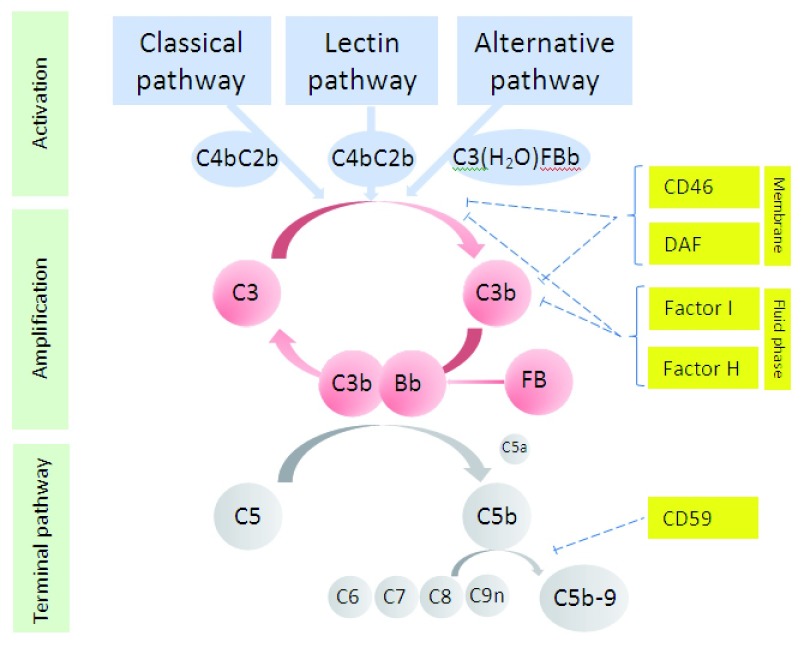
Activation of the complement cascade. DAF, decay accelerating factor.

***Pathogenesis***. The complement system is a complex network of over 40 proteins that constitutes a major part of the innate immune system and contributes to the control of the adaptive immune response. Complement is activated through three distinct pathways ( Figure 1). The classical pathway is initiated when C1q binds immunoglobulin (IgG and IgM) or other non-immunoglobulin moieties. The lectin pathway is activated when pathogen-associated carbohydrates are recognised by mannose-binding lectin, ficolins, or collectins. The alternative pathway exists in a continuous state of low-level activation in order to respond rapidly to infection but also has an important role in the amplification of the classical and lectin pathways. All pathways converge with proteolytic cleavage of C3, producing C3a and C3b and initiating the terminal complement pathway. This leads to the generation of C5a and assembly of the membrane attack complex, C5b-9, two of the main effectors of complement function. The potential for complement amplification means that tight control is essential to prevent excessive activation, and this is provided by a series of soluble and membrane-bound inhibitors.

Complement Factor H (CFH) is a fluid-phase complement inhibitor with both decay-accelerating and cofactor activity. Inherited defects in Factor H function were the first complement abnormalities associated with atypical HUS ^43^. The gene for *CFH* exists in a gene cluster (Regulators of Complement Activation cluster) along with five highly homologous genes encoding the Factor H-related proteins ( *CFHR1–5*). It is now known that mutations, deletions, and genomic rearrangements are common in this region and variants in *CFH* are the most common disease-causing variants (20–30%) ^44– 46^. Mutations in the serine protease Factor I (5–10% of cases) ^47, 48^ and Membrane Cofactor Protein (MCP, CD46, 10% of cases) ^49, 50^ are also associated with disease. In addition, an acquired defect in complement control due to autoantibodies against Factor H, usually associated with homozygotic deletion of *CFHR1–3*, can also cause atypical HUS ^51, 52^, as can gain-of-function mutations in proteins involved in the activation of complement, including variants in C3 (5% of cases) ^53^ and rarely Factor B ^54^. Distinguishing disease-causing mutations from variants of uncertain significance or benign polymorphisms is complex and requires the combination of expert knowledge and application of international standards ^55^. Not all patients who carry a mutation develop disease, perhaps 50–60%, with commonly occurring genetic polymorphisms in *CFH* and *CD46* ^56^ modifying this risk and an environmental trigger required to precipitate disease.

***Diagnosis and management***. Diagnosis is primarily clinical and based on the exclusion of other possible causes of TMA, particularly TTP and STEC infection. C3 can be low in this patient group, but a normal C3 level does not exclude complement-mediated atypical HUS. Genetic screening, assessment for the presence of autoantibodies, and neutrophil surface CD46 levels should be performed in all patients. However, these results, particularly genetic analysis, are not immediately available and therefore do not guide initial diagnosis and early treatment choices.

Plasma exchange was the standard treatment and is still first line until TTP has been excluded (preserved ADAMTS13 activity). Once TTP has been excluded, eculizumab is currently the optimal treatment, but because of its high price this is not available in all countries and plasma exchange is still used. Eculizumab is a highly effective treatment for complement-mediated atypical HUS, establishing remission in >85% of cases ^57^ with a significant improvement in renal outcomes ^58^. The current licence for eculizumab recommends continued treatment because of the risk of relapse. However, several series have now reported withdrawal of eculizumab in patients with complement-mediated disease, with the risk of relapse particularly low in those patients without a mutation ^59– 61^. There is a risk of relapse, particularly in patients with Factor H mutations, but re-introduction of eculizumab rapidly re-establishes remission. The use of complement inhibition to induce remission and intermittently treat relapse may be a future strategy to manage disease that would reduce the treatment burden, cost, and infection risk associated with continuous treatment. As the liver is the main site of complement protein synthesis (with the exception of CD46), liver transplantation has been proposed as a cure for genetic causes of complement-mediated atypical HUS, particularly in those patients who also require a kidney transplant ^62^. With the availability of eculizumab, this option is used less frequently.

For patients with complement-mediated atypical HUS who develop ESRD, there is high risk (80–90%) of recurrent disease following transplantation ^63, 64^. This is true for all genetic and acquired causes of aHUS with the exception of patients who have a mutation in *CD46*, which, because it is expressed on the renal transplant endothelium, has normal function in the transplant kidney. Disease can recur in patients in whom no defect in complement regulation is found, although the risk is lower (30%) ^65^. Recurrence usually occurs early (within 3–6 months) and is associated with a high risk of graft loss ^64^. With the availability of eculizumab, patients can now be successfully transplanted, with options to use eculizumab to treat disease recurrence or prophylactically to prevent recurrence ^66^.

### Other inherited disorders associated with atypical HUS

Homozygous or compound heterozygous mutations in diacylglycerol kinase ε can lead to atypical HUS ^67^. The mechanism by which this lipid kinase leads to a TMA is unknown, but a number of possible mechanisms have been suggested, including release of pro-thrombotic factors, platelet activation, and changes in vascular tone. Patients usually present in childhood, significant proteinuria is common, there may be histological features of membranoproliferative glomerulonephritis ^68^, and the disease may follow a relapsing–remitting course ^69^. Patients commonly progress to CKD and ESRD, at which stage transplant is an option, as disease recurrence has not been reported ^69^. There is no effective treatment and, as complement is not involved in disease development ^70^, eculizumab does not control disease ^67, 69^.

Methylmalonic aciduria and homocystinuria type C protein is involved in the metabolism of vitamin B12 (cobalamin). Homozygotic or compound heterozygotic mutations in the gene encoding this protein, *MMACHC* (cobalamin C [cblC] defect), causes an atypical HUS-like disease ^71^ in addition to a wide range of other clinical features including neurological, cardiac, and pulmonary abnormalities (particularly pulmonary hypertension) ^72^. Biochemically, patients have elevated plasma homocysteine, low methionine, and methylmalonic aciduria. Biochemical and genetic analysis is vital in patients presenting with a TMA, particularly if there are other clinical abnormalities. Patients usually present in childhood, and without treatment the prognosis is poor, with a 100% mortality reported without treatment ^72^. Patients respond to treatment with hydroxocobalamin (B12) and betaine, and treatment can be initiated before the results of biochemical and genetic analyses are available ^73^. Complement is unlikely to have a role in this disease, and experience suggests that eculizumab is ineffective ^74^.

Genetic variants in thrombomodulin ( *THBD*) have been reported in patients with atypical HUS ^75^. Thrombomodulin is part of the coagulation pathway and enhances thrombus formation. It may also activate complement and possibly links these two pathways. New genes associated with atypical HUS are being identified, for example *INF2* ^76^, which may provide insights into the pathophysiology of TMA as well as explain the disease in some patients in whom no complement defect is identified.

## Atypical HUS occurring in specific clinical scenarios

A TMA can develop in a range of different scenarios and, whilst in some cases, as in pregnancy-associated TMA, patients may have a defect in complement regulation, in most cases no abnormality will be found ^77^.

### Pregnancy-associated TMA

There are a number of causes of a TMA either during pregnancy or in the post-partum period, including pre-eclampsia, HELLP syndrome, TTP, and atypical HUS. Differentiating among these can be difficult and is reviewed elsewhere ^78^. Approximately 20% of cases of atypical HUS occur in association with pregnancy, and these most commonly occur in the post-partum period ^79^. A significant proportion of women developing atypical HUS in association with pregnancy have a genetic defect in a complement regulator, suggesting pregnancy is the trigger for disease in women with a genetic predisposition ^79, 80^. These observations have led to the use of eculizumab in pregnancy-associated HUS with reports of good outcomes ^81^, although no trial data are available.

### TMA associated with severe hypertension

Severe hypertension causing a TMA and atypical HUS due to an inherited defect in complement regulation can present with identical clinical features. Pre-existing hypertension, particularly if poorly controlled, and other features of hypertensive end organ damage make a secondary TMA more likely. However, if there is no improvement in laboratory parameters with control of blood pressure, treatment with eculizumab should be considered at least until genetic analysis is available. This strategy avoids missing patients with a defect in complement regulation rather than suggesting that hypertension-mediated TMA will respond to complement inhibition ^82^.

### Drug-related TMA

The development of a TMA has been reported in association with the use of a range of drugs including those used in the treatment of cancer ^83^, immunomodulators, and anti-platelet drugs ( Table 2). For some drugs, this appears to be a direct effect on the endothelium, as is the case for interferon-β ^84^ and bevacizumab ^85^, whilst in the case of quinine the TMA is due to the development of autoantibodies against platelet glycoproteins. The TMA induced by clopidogrel ^86^ and ticlopidine ^87^ leads to the production of antibodies against ADAMTS13 and a TTP-like disease that responds to plasma exchange. In most cases, the exact mechanism of TMA development is unknown and management is supportive with withdrawal of the causative drug.

**Table 2.** Drugs implicated in the development of a thrombotic microangiopathy.

Chemotherapy drugs
**Cisplatin** **Mitomycin** **Gemcitabine** **Vincristine** **Oxaliplatin** **Pentostatin**
Anti-platelet drugs
**Clopidogrel** **Ticlopidine**
Vascular endothelial growth factor inhibitors
**Bevacizumab** **Ramucirumab** **Aflibercept**
Immunosuppressive drugs
**Ciclosporin** **Tacrolimus** **Sirolimus** **Everolimus** **Alemtuzumab (Campath)** **Muromonab-CD3**
Interferons
**Interferon-α** **Interferon-β**
Tyrosine kinase inhibitors
**Sunitinib** **Sorafenib** **Cediranib**
Antibiotics
**Penicillin** **Ciprofloxacin** **Sulfisoxazole**
Illicit drugs
**Cocaine** **Heroin** **Ecstasy**
Miscellaneous drugs
**Oral contraceptive pill** **Quetiapine**

### Malignancy-related TMA

TMAs can occur in a wide range of malignancies including stomach, breast, and bowel cancer and haematological malignancy ^88^. The exact mechanism of disease is unknown, but microvascular metastases may initiate the TMA. In some patients, it may be difficult to differentiate between a TMA caused by the malignancy or due to the chemotherapy used in its treatment.

### Post bone marrow transplant TMA

The development of a TMA has been reported in 10–40% of patients following allogeneic bone marrow transplantation. There are several factors that could contribute to this, including the graft-versus-host response, calcineurin inhibitors, chemotherapy, and infection. Defects in complement regulation have also been reported ^89^, and there is evidence for complement activation (increased soluble C5b-9) in some cases. These observations have led to the use of eculizumab in this situation ^90, 91^, although evidence that this should be part of the management of this condition is still lacking.

### TMA following solid organ transplantation

A TMA can occur after any solid organ transplant, most frequently kidney transplantation, and is due to a number of factors including calcineurin inhibitor toxicity, ischaemia reperfusion injury, antibody-mediated rejection, and infection. Complement mutations have been reported, in up to 30% from one series ^92^, possibly due to recurrence of previously undiagnosed atypical HUS. Complement inhibition should be considered, particularly if there is uncertainty about primary diagnosis or the TMA does not resolve with measures such as calcineurin inhibitor withdrawal.

### TMA occurring in patients with autoimmune disease

The presence of a TMA has been reported in association with a range of primary glomerular diseases, for example, IgA nephropathy, ANCA-associated vasculitis, and FSGS. Patients with C3 glomerulopathy, a disease also characterised by complement dysregulation, may also develop a TMA ^93^. A TMA can also develop in lupus nephritis and in patients with antiphospholipid syndrome (APS), particularly catastrophic APS. Complement is activated in both of these diseases and a role for eculizumab has been reported ^94^, but evidence from clinical trials is not available. A TMA can also be seen in patients with scleroderma renal crisis.

## Concluding remarks

HUS can develop in response to a number of different triggers and in a variety of clinical situations. Identifying the cause of HUS can be difficult but is critical, as the treatment and prognosis is dependent on accurate and timely diagnosis.
